# Antibody dynamics in Japanese paediatric patients with influenza A infection treated with neuraminidase inhibitors in a randomised trial

**DOI:** 10.1038/s41598-019-47884-0

**Published:** 2019-08-15

**Authors:** Nobuo Hirotsu, Yutaka Saisho, Takahiro Hasegawa, Mitsutaka Kitano, Takao Shishido

**Affiliations:** 1 Hirotsu Clinic, Kawasaki, Japan; 2 Shionogi & Co., Ltd., Osaka, Japan

**Keywords:** Influenza virus, Influenza virus

## Abstract

Neuraminidase inhibitors (NAIs) complement influenza virus infection management by helping to clear virus, alleviate symptoms, and reduce transmission. In a previous randomised study, we examined the effect of 4 NAIs on virus clearance and influenza symptoms in Japanese paediatric patients. In this second analysis, we examined the effects of NAI treatment on antibody responses and virus clearance, and the relationships between antibody responses and patients’ infection histories (previous infection; asymptomatic infection via household members of same virus type/subtype; vaccination), and between infection histories and viral kinetics. Haemagglutination inhibition (HI) antibody responses produced HI titres ≥40 by Day 14 of NAI treatment, in parallel with virus clearance (trend test *P* = 0.001). Comparing patients with and without influenza infection histories (directly or asymptomatic infection via household members) showed that infection history had a marked positive effect on HI antibody responses in patients vaccinated before the current influenza season (before enrolment). Current virus clearance was significantly faster in patients previously infected with the same virus type/subtype than in those not previously infected, and clearance pattern depended on the NAI. Assessment of anti-influenza effects of antiviral drugs and vaccines should consider virus and antibody dynamics in response to vaccination and natural infection histories.

## Introduction

Influenza infections are responsible for a substantial burden on individuals, communities, and public health worldwide, with children being among the most immunologically vulnerable groups^1–3^. Typically, influenza infections are transient; viral loads peak within 1 to 3 days of infection and decrease over the following 3 to 5 days^4^. The host response to influenza infection starts with an immediate, nonspecific, immune response that is primarily responsible for virus clearance^5^. After antigen presentation involving dendritic cell/T cell interactions, in the mid-to-later stages of infection, the humoral response is activated via B cells, generating protective influenza virus-specific antibodies^6,7^.

**Figure 1:**
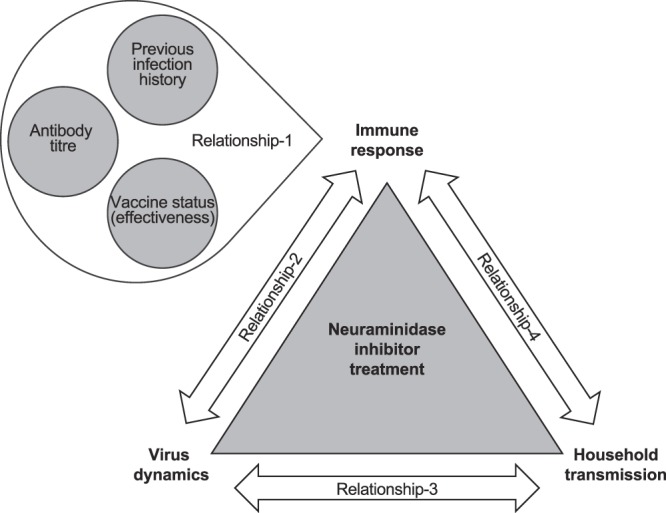
Behaviour of virus, the patient’s immune response to infection, and household transmission are considered to be inter-related in influenza studies. This study is focused on the relationship between patients’ immune response and the immunological factors that may contribute to this response (Relationship-1) and the relationship between patients’ immune response and virus dynamics (Relationship-2).

Neuraminidase inhibitors (NAIs), which help clear influenza virus and have virustatic effects, hasten the alleviation of symptoms and reduce household transmission compared to non-treatment or placebo^8–10^ and, therefore, complement management of infection among individuals and their families. Interpretations of the clinical course of influenza and the effects of NAIs on recovery from influenza infection require an understanding of antibody dynamics over multiple influenza seasons (in response to previous infections, vaccine status and the number of vaccines received, and pre-existing antibody levels), and the relationship between these immune responses (induced by influencing factors) and viral kinetics^11^ (Fig. 1). Although many studies have examined the relationship between vaccination and antibody responses^12–14^, very few have examined the relationship between viral kinetics and antibody responses in animals^15–17^ or humans^18^, or the effects of previous infection history on antibody responses^19–22^.

Among the factors that may influence patients’ immune background, previous infection with whole virus is considered to have the greatest influence. However, in general, tracking of patients’ infection histories is difficult because the duration of infection is short and because patients may visit multiple outpatient clinics between seasons. Because of Japan’s universal health insurance system, most Japanese patients visit a clinic within 48 hours of onset of influenza symptoms, much earlier than in most other countries^23^. In addition, most patients and their families visit the same clinic, visit a clinic known for expertise in influenza treatment, and are prescribed NAIs, which are approved for outpatients in Japan^10^. As such, investigation of the relationships between antibody responses over multiple influenza seasons and in response to a current infection with the same virus type/subtype is possible. At the Hirotsu Clinic, investigation of household transmission over 6 influenza seasons^9^ was conducted in parallel with the current study. Together these studies are allowing a detailed analysis of patients’ previous infection and influenza exposure histories.

The first part of this study examined the effect of 4 NAIs on the primary end point, time to virus clearance (virus titre), and has been reported previously^10^. This second part aimed to determine the effect of NAI treatment on antibody responses (haemagglutination inhibition [HI] titre). In addition, in our cohort of paediatric patients with and without a previous history of influenza infection, we aimed to explore the effect of patients’ immune background (vaccination status, previous influenza infection, or previous asymptomatic infection via household members) on the HI antibody response to a current infection, and the relationship between patients’ immune background and viral kinetics.

## Results

### Patient population

As described previously^10^, baseline characteristics were well balanced among the treatment groups. There were 114 patients (peramivir n = 28, oseltamivir n = 30, zanamivir n = 26, and laninamivir n = 30) in the full analysis set; 91 had influenza A/H3N2 subtype infection (including 89 with both antibody and positive virus titre measurement at baseline), 18 had influenza A/H1N1pdm09 infection, 1 had influenza B infection, and 1 had influenza A and influenza B co-infections. Three patients were PCR negative for influenza virus infection.

### Effect of NAI treatment on antibody dynamics

**Figure 2:**
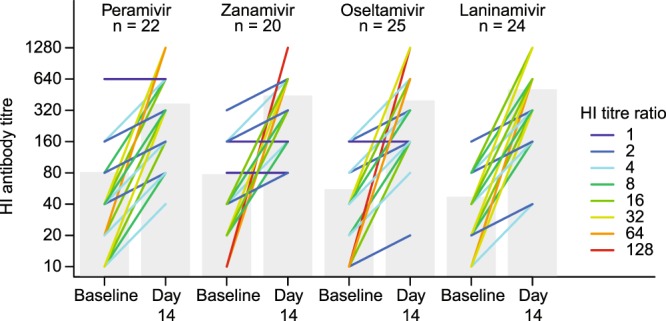
Haemagglutination inhibition (HI) titres against influenza A/H3N2 vaccine strains at baseline and Day 14 by neuraminidase inhibitor treatment. Coloured lines show HI titre ratios for the change in HI titre from baseline to Day 14 for individual patients. Columns show mean change at each time point. One patient had HI titre < 40 at Day 14. HI titres < 10 were treated as 10 in the statistical analyses.

At Day 14, HI titres remained at ≥40 or rose to ≥40 in all but 1 patient whose antibody levels remained low (ie, <40) at baseline and Day 14 with an influenza A/H3N2 infection (Fig. 2). There were no notable differences in the change in mean HI titres from baseline to Day 14 among the NAIs (Fig. 2). For individual patients, the increase in HI titre by Day 14 was smaller for those with higher HI titres at baseline.

**Figure 3:**
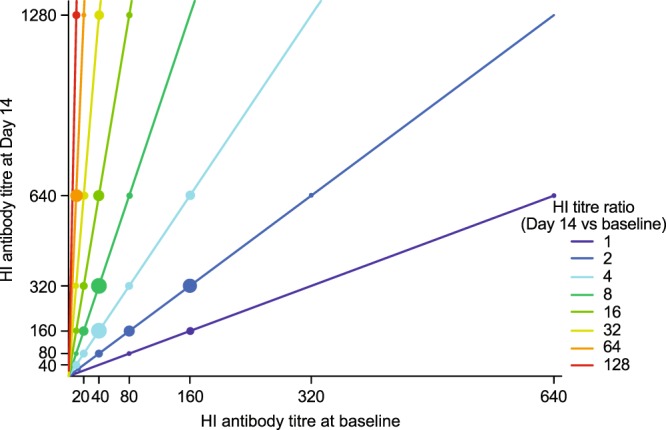
Scatter plot of haemagglutination inhibition (HI) titres against influenza A/H3N2 infection at baseline vs HI titres at Day 14. The colour of each line shows the HI titre ratio at Day 14 relative to the baseline levels and the size of the coloured circles reflects the patient number at each point. HI titres < 10 were treated as 10 in the statistical analyses.

We further explored the relationship between baseline (pre-existing) HI titres and the increase in HI titres during infection (Fig. 3). Patients with low baseline HI titres had greater HI antibody responses at Day 14 than patients with high HI baseline titres. HI titre ratios at Day 14 relative to baseline were ≥16 for 48% (29/60) of patients with baseline HI titres <80 and were ≤8 for 93% (27/29) of patients with baseline HI titres ≥80 (Fig. 3).

HI antibody titres during influenza infection did not change substantially from baseline to Day 3, but increased approximately 7-fold from baseline (mean HI titre = 64) to Day 14 (mean HI titre = 431) for influenza A/H3N2, and approximately 14-fold from baseline (mean HI titre = 35) to Day 14 (mean HI titre = 484) for influenza A/H1N1pdm09 (Supplementary Fig. S1).

### Patients’ previous exposure to influenza virus

For influenza A/H3N2 infection, vaccination history was assessed in patients with a previous influenza A/H3N2 infection and in patients with a previous influenza A/H3N2 infection within their household (Table 1). Of the 29 patients with confirmed previous influenza A/H3N2 infection, 18 had been vaccinated and 11 had not. Of the 12 patients with a confirmed household history of influenza A/H3N2 infection, 9 had been vaccinated and 3 had not. No patient had a previous influenza A/H1N1pdm09 infection. The effect of vaccination history in patients with influenza A/H1N1pdm09 infection was not investigated further because of the small number of patients (Table 1).

**Table 1:** Previous infection and vaccination status in enrolled patients just before the influenza season

Infection status	n	Vaccination history (n)	
		None	Any (1, 2)
**Previous A/H3N2 infection: patient**			
No	78	37	41 (14, 27)
Yes	29	11	18 (3, 15)
Total	107	48	59 (17, 42)
**Previous A/H3N2 infection: household member, not patient**			
No	55	29	26 (10, 16)
Yes	12	3	9 (4, 5)
Total	67	32	35 (14, 21)
**Previous A/H1N1pdm09 infection: patient**			
No	16	7	9 (0, 9)
Yes	0	0	0 (0, 0)
Total	16	7	9 (0, 9)

Note: only patients infected with influenza were enrolled in this study, resulting in possible selection bias with the exclusion of non-infected individuals.

### Effect of patient immune background on antibody dynamics

**Figure 4:**
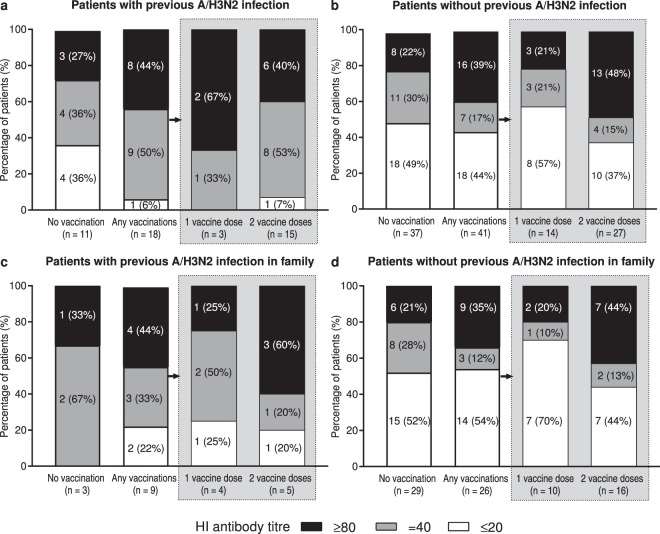
Pre-existing haemagglutination inhibition (HI) titre levels according to vaccination status in patients with (**a**) and without (**b**) previous influenza A/H3N2 infection and in patients with (**c**) and without (**d**) previous household member infection. Columns indicate the percentage of each subgroup with HI titre ≤ 20 (white), = 40 (grey), or ≥ 80 (black); number of patients in each HI titre category are shown within each column.

The potential positive effects of patient exposure to influenza virus on pre-existing HI antibody titres after vaccination were assessed in patients with a previous influenza A/H3N2 infection and in patients with a previous influenza A/H3N2 infection history within their household (Fig. 4). One vaccine dose resulted in HI titres ≥40 in most patients with a previous history of influenza A/H3N2 infection (directly or by asymptomatic infection). The percentage of patients with HI titres ≥40 was 100% (3/3) and 93% (14/15) after 1 and 2 vaccine doses, respectively, in patients who had been infected directly (Fig. 4a), and was 75% (3/4) and 80% (4/5) after 1 and 2 vaccine doses, respectively, in patients with a household history of influenza A/H3N2 infection (Fig. 4c). However, the number of patients with 1 or 2 doses of vaccine in both groups, and the number of patients in all subgroups of patients with a household history of influenza A/H3N2 infection, was small.

The effect of vaccination on pre-existing HI titres was lower in patients without a previous infection history (either directly or by asymptomatic infection) compared to patients with previous infection histories (Fig. 4). The percentage of patients with HI titres ≥40 was 43% (6/14) and 63% (17/27) after 1 and 2 vaccine doses, respectively, in patients without a previous influenza A/H3N2 infection (Fig. 4b), and was 30% (3/10) and 56% (9/16) after 1 and 2 vaccine doses, respectively, in patients without a household history of influenza A/H3N2 infection (Fig. 4d).

We further explored the effect of patient infection history on HI antibody titres among patients with a previous influenza A/H3N2 infection for which the timing of that infection was known (n = 21 patients). There was no apparent decrease in HI titre with time for up to 4 years (Supplementary Fig. S2).

### Effect of the presence of virus on antibody dynamics over time in patients with previous influenza A/H3N2 infection

**Figure 5:**
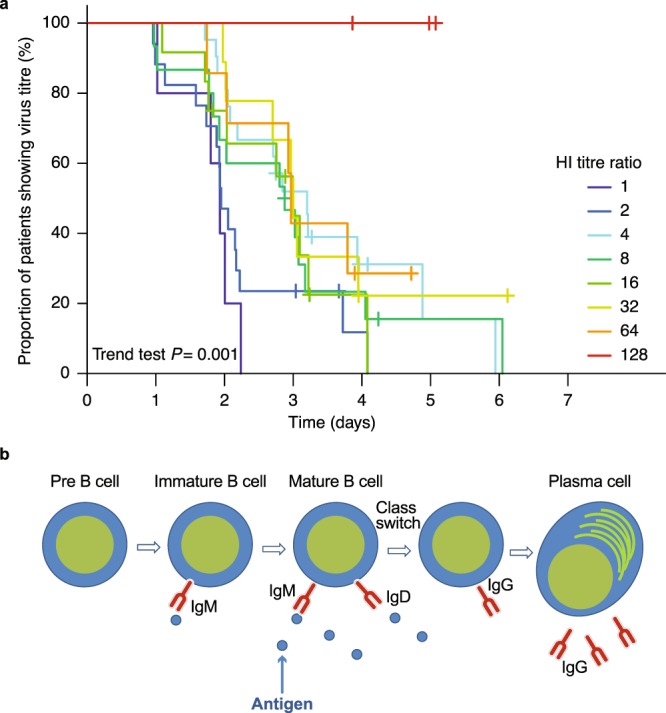
(**a**) Kaplan–Meier curve of time to influenza A/H3N2 virus clearance based on the magnitude of increased haemagglutination inhibition (HI) titre from baseline to Day 14. The colour of each line shows the ratio of HI titre at Day 14 relative to baseline. HI titres < 10 were treated as 10 in the statistical analyses. HI titre ratio of 1 (80 at baseline for 1 patient, 160 for 3 patients, and 640 for 1 patient), 2 (10 at baseline for 1 patient, 40 for 3 patients, 80 for 5 patients, 160 for 7 patients, 320 for 1 patient), 4 (10 at baseline for 3 patients, 20 for 3 patients, 40 for 8 patients, 80 for 3 patients, and 160 for 4 patients), 8 (10 at baseline for 1 patient, 20 for 4 patients, 40 for 8 patients, and 80 for 2 patients), 16 (10 at baseline for 2 patients, 20 for 3 patients, 40 for 5 patients, and 80 for 2 patients), 32 (10 at baseline for 2 patients, 20 for 3 patients, and 40 for 4 patients), 64 (10 at baseline for 6 patients and 20 for 1 patient), 128 (10 at baseline for 3 patients) (**b**) B cell maturation and flow. At the beginning of a primary infection, immature B cells provide a short-lived antibody (immunoglobulin M [IgM]) response, independent of antigen. In the later stages of infection, and after antigen presentation involving dendritic cell/naïve T cell interactions in the lymph node, T cells interact with naïve B cells, which then cluster and mature in the germinal centre to produce a longer-lived and antigen-dependent antibody response (immunoglobulin G [IgG]) comprising antibody-secreting plasma cells and memory B cells.

There was a clear relationship (trend test *P* = 0.001) between influenza A/H3N2 virus clearance during the first week after the start of NAI treatment and the resulting HI antibody titre at Day 14 relative to baseline (Fig. 5). Patients who cleared virus more slowly had high HI titres at Day 14 relative to baseline (HI titre ratio ≥4), whereas patients who cleared virus more quickly had low HI titres at Day 14 relative to baseline (HI titre ratio 1 or 2). In addition, the rate of virus clearance appeared to plateau in patients with high HI titres (≥40) at baseline (Supplementary Table S1). The median number of days in which patients were positive for influenza A/H3N2 virus was longer by approximately 1 day in patients with low baseline HI antibody titres (<40) compared with patients with high baseline HI titres (≥40) (Supplementary Table S1). There were no differences in clinical efficacy end points such as time to resolution of fever and time to alleviation of symptoms among the 4 NAI treatment groups, although there were differences in the pattern of viral clearance between peramivir and oseltamivir^10^.

### Effect of patient background on viral kinetics in patients with influenza A/H3N2 infection

**Figure 6:**
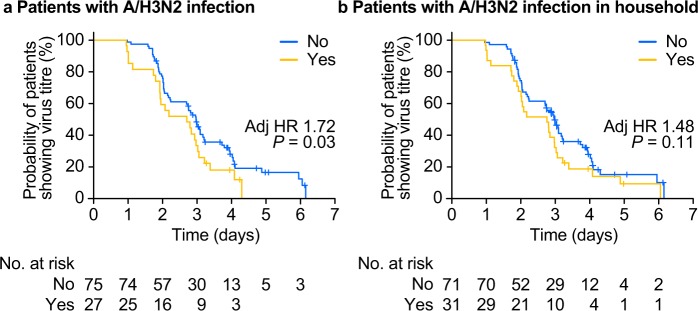
Kaplan–Meier curves of the time from the start of treatment with neuraminidase inhibitor to influenza virus titre clearance according to the presence (yellow line) or absence (blue line) of previous influenza A/H3N2 infection in (**a**) the patient or (**b**) a household member. Adj, adjusted; HR, hazard ratio.

After NAI treatment, virus was cleared significantly more rapidly in patients with previous influenza A/H3N2 infection than in patients without previous infection (*P* = 0.03; Fig. 6a). Virus was also cleared more rapidly in patients with a household history of influenza A/H3N2 infection than in patients without a household history of infection, but the difference was not significant (adjusted hazard ratio [HR]: 1.48 [95% CI, 0.92 to 2.39]; *P* = 0.11) (Fig. 6b).

**Figure 7:**
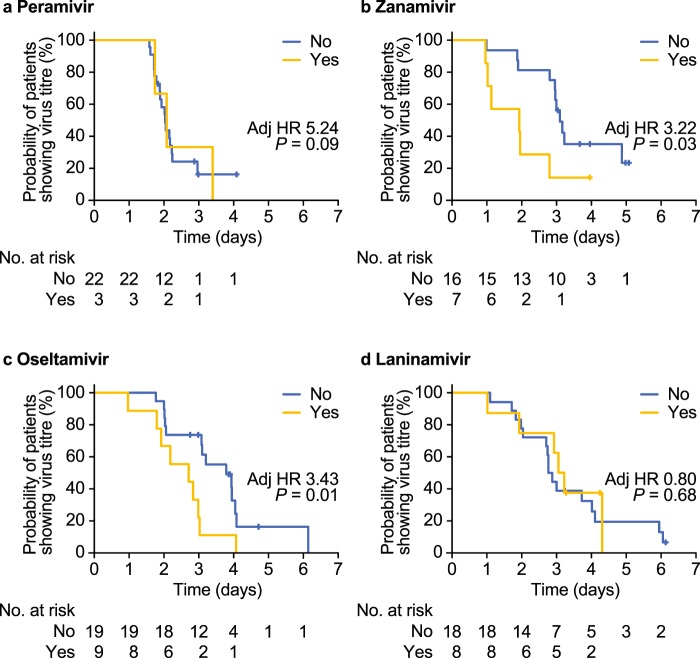
Kaplan–Meier curves of the time from the start of treatment with a neuraminidase inhibitor to influenza virus titre clearance according to the presence (yellow line) or absence (blue line) of previous influenza A/H3N2 infection: (**a**) peramivir, (**b**) zanamivir, (**c**) oseltamivir, or (**d**) laninamivir. Adj, adjusted; HR, hazard ratio.

The virus clearance pattern for patients with a previous A/H3N2 infection differed between NAIs (Fig. 7a,d). For peramivir (adjusted HR: 5.24 [95% CI, 0.79 to 34.71]; *P* = 0.09) and laninamivir (adjusted HR: 0.80 [95% CI, 0.29 to 2.26]; *P* = 0.68), the difference in time to virus clearance between patients with and without previous influenza A/H3N2 infection was not significant. As reported previously^10^, virus clearance was more rapid with peramivir than with laninamivir, even though a similar clearance pattern was observed (Fig. 7a,d). For zanamivir (adjusted HR: 3.22 [95% CI, 1.09 to 9.50]; *P* = 0.03) and oseltamivir (adjusted HR: 3.43 [95% CI, 1.32 to 8.88]; *P* = 0.01), the time to virus clearance was significantly more rapid in patients with previous influenza A/H3N2 infection than in patients without previous infection (Fig. 7b,c).

## Discussion

In this study of paediatric patients with influenza infection, HI titres increased to ≥40 in most patients by Day 14 of NAI treatment for a current influenza infection, which is consistent with the antibody response generally accepted as the level corresponding to a 50% decreased risk of influenza in adults^24^. However, children have immature immune systems because of their lower level of exposure to seasonal influenza viruses compared with adults, and no HI titre cut-off point for children has been universally accepted^24^. A placebo-controlled arm was not included in this study because it was primarily designed to compare NAIs in clinical practice where all paediatric patients receive treatment. Despite this, the antibody response observed occurred in parallel with a decrease in virus during NAI treatment, which is consistent with placebo-controlled studies in adults showing that NAIs do not negatively affect the antibody response^25,26^ and a study in paediatric patients showing positive antibody responses after treatment with a single peramivir injection approximately 5 days after influenza virus infection^27^.

As an exploratory study, the main focus here was to assess antibody dynamics in NAI-treated patients with the commercial HA antigens derived from A/H3N2 and A/H1N1pdm09 influenza vaccine strains that were used over 2 influenza seasons (2013–2015). The HA antigen is responsible primarily for virus attachment and entry into host cells, and is the major target for development of influenza vaccines^5^. In Japan, the current influenza vaccines are either split vaccines (ie, contain both extracted HA and neuraminidase [NA]) or subunit vaccines (ie, predominantly composed of HA). However, circulating influenza strains are whole viruses that contain intact NA and HA antigens together on the surface of the virus. Recently, the A/H3N2 influenza virus has undergone an antigenic drift, resulting in NA-receptor variants that affect HA activity and the ability of anti-HA antibody to inhibit virus attachment to host cells^28^. As a consequence, HI titres against the HA antigen in patient-derived A/H3N2 influenza virus in this study would have been affected and, therefore, were not measured. In addition, we believe that the confounding effects of NA-mediated haemagglutination in our study are likely to be minimal because the effect of NA-receptor variants on HA activity is not evident with the commercially available HA antigen used (data not shown). The A/H1N1pdm09 influenza virus had not undergone antigenic changes during this study, which was confirmed by the patients (n = 18) who were newly infected with influenza A/H1N1pdm09 (ie, they did not have a history of previous infection with the same virus type/subtype as A/H1N1pdm09).

Findings from 2 key studies investigating the effects of patients’ infection history on the antibody response to influenza infection suggest that patients’ responses are more complex than originally thought, and comprise a continual accumulation and degradation of HI antibody responses over time, boosting of responses to historical infections with older strains of influenza virus, and cross-reactivity^19,21,22^. However, these studies are limited because they rely on estimates of infection histories from cross-sectional serological data to develop models of HI antibody profiles for individual patients over time and across infection seasons. In this study, we assessed the relationship among antibody dynamics, viral kinetics, and previous exposure to virus or virus antigen (by previous infection, asymptomatic infection, or vaccination) in the same cohort of paediatric patients and their families in a broad sense. By comparing HI antibody responses between patients with and without a history of influenza A/H3N2 infection, we showed that a previous influenza A/H3N2 infection had a marked positive effect on the HI antibody response in patients who had been vaccinated before the current influenza season (ie, before their enrolment in the study). In contrast to vaccinated patients without previous infection, more than 90% of vaccinated patients with a previous influenza A/H3N2 infection of the same type/subtype had an HI titre ≥40, and only 1 dose of vaccine was sufficient to elicit this response. Moreover, HI antibody titres in response to previous influenza A/H3N2 exposure were similar among infected patients and those exposed to an infected household member, suggesting that even asymptomatic infection may be sufficient to elicit an antibody response equivalent to natural infection in previously vaccinated patients. Although we did not measure directly the change in pre-existing antibody titre between infections among the patient cohort, there did not appear to be a decrease in pre-existing HI titres over time (Supplementary Fig. S2), which further supports the long-term effect of previous influenza infections on patients’ immune profiles. Furthermore, the degree of antigenic differences reported among circulating A/H3N2 influenza strains from 2010–2015 in Canada^29^, which are similar to the strains circulating in Japan, suggests that the antigenic drifts between a previous and current infective strain are likely to produce cross-immunogenicity that also affects virus dynamics.

By confirming antibody dynamics and viral kinetics in the same cohort of paediatric patients, we also showed a more clear-cut correlation between the presence of virus and antibody dynamics than has been reported previously^15–18^. Patients who cleared virus more slowly had greater increases in HI titres at Day 14 compared with those who did not, which suggests that longer exposure to influenza virus results in higher antibody responses, mostly likely because of greater exposure of B cells to virus (Fig. 5b). Moreover, the finding that the median number of virus-positive days appeared to plateau in patients with pre-existing HI titres ≥40 provides further support for this cut-off point as a marker of protection from influenza infection in children (Supplementary Table S1). In essence, the antibody dynamics in response to a current influenza infection observed in our study was consistent with the exposure to virus and with the influence of patients’ immune histories in response to the same virus type/subtype. Following NAI treatment, clearance of virus was significantly faster in patients with previous exposure to A/H3N2 (via previous infection). As expected, the higher levels of pre-existing antibodies observed in these patients would allow these individuals to eliminate a subsequent infection more effectively than infection-naïve individuals. The mechanism underlying this effect is distinct from typical antibody cross-reactivity and is thought to be driven by memory B cells generated during patients’ previous exposure to A/H3N2 influenza that generate a rapid and broadly reactive antibody response during subsequent infections^22,30^. Virus clearance was also more rapid in patients who had a household history of influenza infection, which is consistent with a rapid memory B cell response at the site of infection^30^, and which suggests that even asymptomatic infection via household members is sufficient to generate this response. However, the analyses for household infection comprised a small number of patients and the finding for virus clearance was not statistically significant. In addition, we did not include patients without a current infection in this study as a control. Therefore, we cannot confirm whether higher pre-existing antibody levels arising from previous infection can confer protection.

The different patterns of viral clearance among the NAIs in patients with and without previous A/H3N2 infection in this study are likely to be related to the pharmacodynamic and pharmacokinetic properties of each NAI^31^. The effects of NAI treatment on viral clearance are most evident during the early stages of infection, before the onset of a patient’s immune response in the mid-to-later stages of infection^6^. Consistent with the primary outcome of our study^10^, there was a rapid reduction in virus titre in patients who had received peramivir compared with oseltamivir, and the time to virus clearance did not differ between patients with or without a previous infection in peramivir-treated patients. As peramivir is administered intravenously, onset of activity is more rapid compared with other NAIs^6,32^. Therefore, the lack of difference between patients with and without previous infection is likely because the effects of peramivir on viral clearance appear earlier, before contributions of the antigen-specific antibody response are apparent. Although a similar pattern was observed with laninamivir, the overall time to virus clearance seems to be longer than with peramivir. As laninamivir is a long-acting NAI^33^, its effects are observed during the early-to-mid stages of infection, which would lead to relatively smaller effects on viral clearance than peramivir during the early stages of infection. In patients receiving zanamivir or oseltamivir, the time to virus clearance was significantly shorter in those with previous infection than in those without previous infection. This is likely because the onset of activity of these NAIs occurs in the later stages of disease compared with intravenous peramivir^6^, when antiviral effects are likely to be additive to patients’ immune responses.

The findings from this study have shown the relationships between pre-existing antibody responses to influenza (from direct infection, asymptomatic infection, and vaccination), viral kinetics, and the immune response to infection in the same cohort of patients. However, there are several limitations that should be considered. First, as we only enrolled patients with a current influenza infection, we cannot rule out any potential effects arising from selection bias with the exclusion of non-infected individuals. Second, as previously stated, we did not include a placebo-controlled arm and the sample sizes for the subpopulation comparisons were not powered to show statistically significant differences. Third, as this was an exploratory study, the number and type of measures that could be included were limited to those that were predetermined by the protocol and by the small volume of blood collection allowed. Finally, the limited number of patients with influenza A/H1N1pdm09 prevented an in-depth analysis. None of the 16 patients with A/H1N1pdm09 infection had been infected previously, suggesting that this subtype may have maintained its antigenicity over 4 seasons. However, during the 2015–2016 season, there were patients attending the clinic who had a second infection with A/H1N1pdm09, suggesting more recent antigenic drift^34^.

In summary, by comparing HI antibody responses between patients with and without a history of infection (by direct infection or asymptomatic infection via a household member), we showed that previous influenza virus infection in patients who had been vaccinated before enrolment in the study had a more positive effect on the HI antibody response compared to patients who had not been vaccinated. In addition, virus clearance during a current infection was significantly faster in patients previously infected with influenza virus of the same virus type/subtype than in those not previously infected, and the effects of NAIs on viral clearance differed with the pharmacodynamic and pharmacokinetic properties of each NAI. Together these data suggest that assessment of the anti-influenza activity of NAIs should consider virus and antibody dynamics based on not only authentic vaccination histories but also natural infection histories^21,22^.

## Methods

### Study design

This single-centre, open-label, randomised study (UMIN-CTR UMIN000012670, date of registration Dec 24, 2013), conducted at Hirotsu Clinic, Kawasaki, Japan, enrolled patients from January 2014; final patient follow-up was March 2015 (2 northern hemisphere seasons). The study protocol was approved by the ethics committee of Shionogi & Co., Ltd and the study was undertaken in compliance with the Declaration of Helsinki and the Ministerial Ordinances related to Good Clinical Practice of Japan. All patients and/or their parents/guardians provided written informed consent. Full details of the study design and the primary results of the study have been published previously^10^.

### Study population

As described previously^10^, enrolled patients were 4 to 12 years with influenza A virus infection, were available for treatment within 48 hours of influenza symptoms (ie, first observed body temperature was ≥37.5 °C), had an axillary temperature ≥37.5 °C, and a positive rapid antigen test (RAT; ImmunoACE Flu^®^; Tauns Laboratories, Inc., Shizuoka, Japan). The use of RAT allowed enrolment of patients who did not meet the criteria for the presence of 6 influenza-related symptoms (cough, sore throat, headache, nasal discharge, muscle or joint pain, and fatigue). Influenza infection was confirmed by polymerase chain reaction (PCR) in addition to RAT. The main exclusion criteria were underlying disease likely to affect viral dynamics and any contraindication to NAI use, including use of other antiviral drugs.

### Treatment protocol

Patients were randomised (1:1:1:1) to receive peramivir, oseltamivir, zanamivir, or laninamivir; the dose and administration of each NAI were in accordance with the Japanese package insert. Peramivir was given in single doses to all patients (no patient was administered peramivir for ≥2 days).

### Outcome measures

Patients’ infection histories were confirmed for the previous 3 seasons in patients enrolled during the first season, and for the previous 4 seasons in patients enrolled during the second season of the study. Infection histories of household members, and patient’s vaccination status in the same influenza season and just before the influenza season at enrolment, were derived mostly from medical records, and in a limited number of instances, patient self-reports to confirm data or provide missing information.

Serum antibody levels (HI titres) against haemagglutinin (HA) antigen derived from vaccine strains were assessed at a minimum of 3 visits (before treatment at Day 1, Day 3, at diagnosis of a negative RAT from Days 4 to 7 [optional], and Day 14 ± 3). The HA antigen used was in accordance with the vaccines available in Japan at that time. For A/H3N2 HA antigen, A/Texas/50/2012 [H3N2] was used for patients in the 2013–2014 season and A/New York/39/2012 [H3N2] for those in the 2014–2015 season; A/California/7/2009 [H1N1] pdm09 was used for those in both seasons. HI titre against patient-derived influenza virus was only measured for A/H1N1pdm09 influenza. HI titre against patient-derived A/H3N2 influenza was not measured because the virus had undergone antigenic changes, which would affect HI titre measurement^28^.

### Haemagglutination inhibition (HI) titres and virus titres

Patient-derived influenza virus was isolated from patients and propagated as described previously^10^. Briefly, nasal swabs from both nares were collected from patients at the first visit and stored in a viral transport medium until use. These nasal swab fluids were inoculated into Madin Darby canine kidney (MDCK) cells in 24-well plates and centrifuged at 1000 rpm at room temperature for 30 min. After centrifugation, MDCK cells were washed once with culture medium and then incubated at 33 °C in 5% CO_2_. After 7 days’ culture, cell culture supernatants were collected and re-inoculated into MDCK cells in a 75-cm^2^ flask until a 4+ cytopathogenic effect was observed or for 10 days, whichever came first. Cell culture supernatants were collected from those cells and stored at −80 °C until use.

Sera were treated with receptor destroying enzyme (RDEII, Denka Seiken, Tokyo, Japan) to eliminate inhibitors of nonspecific haemagglutination. Serially diluted sera in a microplate were mixed with 4 HA units of a commercial HA antigen (Denka Seiken, Tokyo, Japan) derived from the corresponding vaccine strain or patient-derived influenza virus for 1 hour at room temperature. The mixture was then incubated with 1% guinea pig red blood cells (Nippon Bio-test Laboratories Inc., Saitama, Japan) for 1 hour at 4 °C. HI titres were expressed as reciprocals of the highest dilution of serum samples that completely inhibited haemagglutination.

Virus titre was measured daily by the RAT (ImmunoACE Flu^®^; Tauns Laboratories, Inc., Shizuoka, Japan) until the RAT was negative^10^.

### Statistical analysis

The full analysis set (FAS) was all randomised patients with ≥1 dose of study drug and at least one efficacy measure (eg, time to virus clearance, time to resolution of fever, time to alleviation of symptoms) after starting NAI treatment. Exploratory analyses included patients with available data in the FAS.

Kaplan–Meier curves (for overall population and by NAI) were constructed for time to virus clearance, according to the presence or absence of previous influenza A/H3N2 infection in patients or their household members. Hazard ratios for virus titre in patients with previous infection versus those without previous infection were estimated using a Cox proportional hazards model adjusted for age, influenza vaccine in the same season, and NAI treatment (only for the overall population). Mean HI titres against A/H3N2 vaccine strains at baseline, Day 3, and Day 14 and time to virus clearance (virus titre) are presented for influenza subtype A/H3N2. HI titre <10 was treated as 10 in the statistical analyses. No imputation was performed for missing data. The log-rank test for trend was used to detect ordered alternatives of time to influenza A/H3N2 virus clearance among HI titre ratio groups. Statistical analyses were performed using SAS^®^, Version 9.2 for Windows (SAS Institute, Cary, NC, USA) and R, Version 3.4.1 for Windows^35^.

## Supplementary information

Supplementary Information

## Data Availability

The data for this study contains personal information that is not suitable for sharing in its current format. Appropriately de-identified datasets for the current study can be made available by the corresponding author upon reasonable request.
