# Supplementary material for: Antibody dynamics in Japanese paediatric patients with influenza A infection treated with neuraminidase inhibitors in a randomised trial
Source: Sci Rep. 2019 Aug 15;9:11891. doi: 10.1038/s41598-019-47884-0 (PMC6695405; doi:10.1038/s41598-019-47884-0)
Supplement: Supplementary file 1 — Supplementary Information [file 41598_2019_47884_MOESM1_ESM.pdf]

supplementary information

Antibody dynamics in Japanese paediatric patients with influenza A infection treated with neuraminidase inhibitors in a randomised trial

Nobuo HIROTSU, MD, PhD

Yutaka SAISHO, PhD

Takahiro HASEGAWA, DPH

Mitsutaka KITANO, PhD

Takao SHISHIDO, PhD

Supplementary Table S1. Period in which patients were positive for influenza A/H3N2 according to baseline HI antibody titre.

Baseline HI titre	Peramivir (n)	Zanamivir (n)	Oseltamivir (n)	Laninamivir (n)	Total (n)	Virus-positive period, median (95% CI) (days)
**<40**	8	7	11	6	32	**3.2 (2.9 to 4.1)**
**=40**	6	6	7	9	28	**2.4 (1.9 to 3.1)**
**≥80**	8	7	7	7	29	**2.2 (1.9 to 2.8)**

CI, confidence interval; HI, haemagglutination inhibition.

Supplementary Figure S1

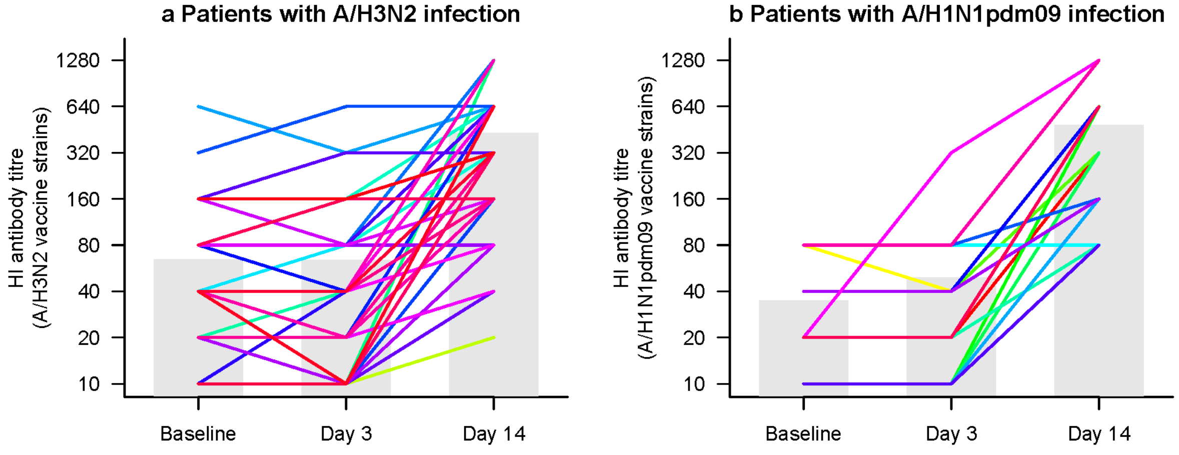

Haemagglutination inhibition (HI) titres at baseline, Day 3, and Day 14 in patients with influenza A/H3N2 infection and A/H1N1pdm09 infection. Each line shows the change in HI titre for an individual patient. The mean at each time point is shown by the columns. HI titres <10 were treated as 10 in the statistical analyses.

Supplementary Figure S2

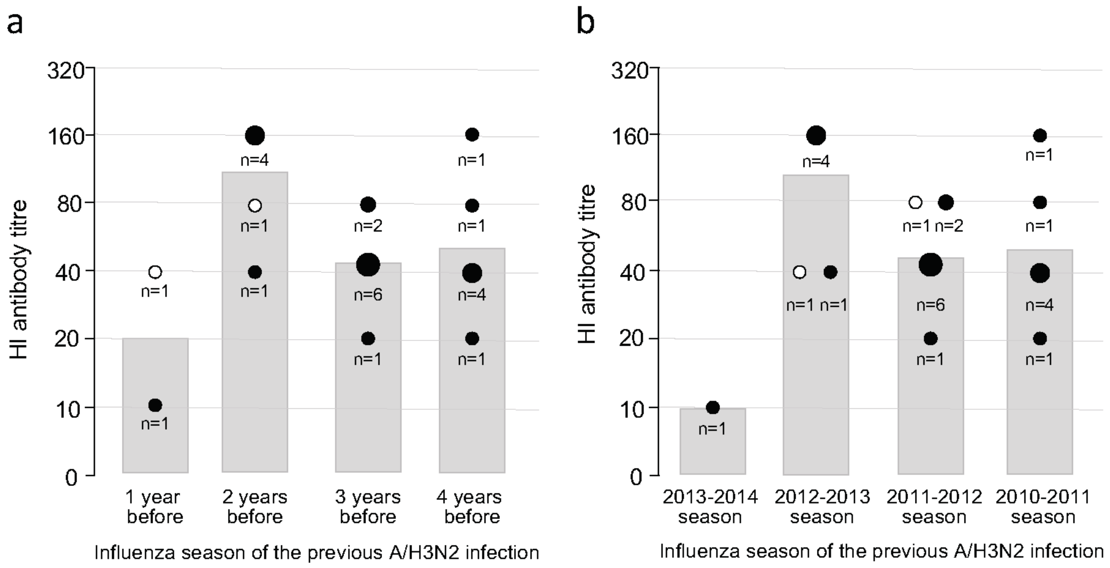

Mean haemagglutination inhibition (HI) titre levels at baseline by (a) time of previous infection and (b) by influenza season of the previous infection. The bubble size indicates the patient number. All patients infected with H3 in the 2014-2015 season were included; patients infected in 2014 (2013-2014 season) are denoted by the open circles.

In both graphs, pre-existing HI antibody titres in patients with a previous infection 3-4 years before their current infection (or in the 2010-2011 and 2011-2012 influenza seasons) were similar. By comparison, HI antibody titres in patients with a previous infection 2 years before their current infection (or in the 2012-2013 influenza season) were slightly elevated. This difference is likely because the vaccine strain for the current season in this study (2014-2015) is the same type/subtype as the circulating virus in the 2012-2013 season (ie, A/New York/39/2012 [H3N2]). Interpretation of the pre-existing HI antibody titres in patients with a previous infection 1 year before their current infection (or in the 2013-2014 influenza season) is limited by the small number of patients.
